# Acute treatment optimization for migraine: results of the American migraine prevalence and prevention (AMPP) study

**DOI:** 10.1186/1129-2377-1-S14-P201

**Published:** 2013-02-21

**Authors:** RB Lipton, AN Manack, D Serrano, DC Buse

**Affiliations:** 1 Department of Neurology, Montefiore Medical Center, Bronx, New York, USA; 2 Allergan, Inc., Irvine, California, USA; 3 Vedanta Research, Chapel Hill, North Carolina, UK; 4 Department of Neurology, Montefiore Medical Center, Bronx, New York, UK

## Objectives

To assess and compare acute treatment optimization as measured by the Migraine Treatment Optimization Questionnaire (M-TOQ) within a population-based sample of persons with migraine.

## Methods

AMPP is a longitudinal, US-population-based study for which questionnaires were mailed to 24,000 severe headache sufferers and followed annually. Respondents with ICHD-2 migraine were stratified as either CM (>15 headache-days/month) or EM (<15 headache-days/month). Acute-treatment optimization was measured with M-TOQ, a valid/reliable patient-report tool assessing 5 domains: functioning, rapid relief, relief consistency, recurrence risk, tolerability over preceding 4 weeks. Respondents rated statements in each area as either occurring: never, rarely, < or > half the time. An item response theory (IRT) model used to define scaled treatment optimization scores as function of M-TOQ item set: lower scores=less/problematic optimization; higher scores=greater optimization. The model was expanded to incorporate persons with CM/EM on scaled scores and explored demographic adjustments for age and gender.

## Results

8612 persons met criteria for migraine (CM=539; EM=8073) and completed M-TOQ. IRT model parameters indicated excellent M-TOQ psychometric properties. Scaled treatment optimization scores were significantly lower for persons with CM (3.25) vs EM (4.01, b=-0.757; p<.0001), corresponding to a 0.5 standard deviation (SD) difference between CM and EM. After adjustment, mean difference on scaled-optimization score remained significantly lower (worse) for CM (b=-0.751; p<.0001).

## Discussion

Treatment regimens were less well-optimized and more lacking in domains measured by M-TOQ (ie, functioning, rapid relief, consistency of relief, risk of recurrence and tolerability) among persons with CM vs EM. Funding: The AMPP study was funded through a research grant to the NHF from Ortho-McNeil Neurologics. Additional analyses were supported by Allergan, Inc.

